# Qingwei San treats oral ulcer subjected to stomach heat syndrome in db/db mice by targeting TLR4/MyD88/NF-κB pathway

**DOI:** 10.1186/s13020-021-00565-5

**Published:** 2022-01-04

**Authors:** Lu Shi, Yongcheng An, Long Cheng, Yiyang Li, Huimin Li, Chen Wang, Yinglan Lv, Yuhui Duan, Hongyu Dai, Changhao He, Huilin Zhang, Yan Huang, Wanxin Fu, ShengPeng Wang, Baosheng Zhao, Yitao Wang, Yonghua Zhao

**Affiliations:** 1 Department of Pharmacology, School of Chinese Materia Medica, Beijing University of Chinese Medicine, Beijing, 102488 China; 2 School of Life Sciences, Beijing University of Chinese Medicine, Beijing, 102488 China; 3 Institute of Chinese Medical Sciences, State Key Laboratory of Quality Research in Chinese Medicine, University of Macau, Research Building N22, Avenida da Universidade, Taipa, Macao SAR, 999078 China; 4 Beijing Research Institute of Chinese Medicine, Beijing University of Chinese Medicine, No. 11 North 3rd Ring East Road, Chao-Yang District, Beijing, 100029 China

**Keywords:** Qingwei San, Oral ulcer, Stomach heat syndrome, Diabetes mellitus, TLR4/MyD88/NF-κB

## Abstract

**Background:**

Qingwei San (QWS), one of classic Chinese Medicine prescripts, has been widely used to treat stomach heat syndrome which manifests oral ulcer (OU), periodontitis and upper gastrointestinal bleeding for seven hundred years. However, the therapeutic effects of QWS on diabetic OU subjected to stomach heat syndrome are still ambiguous. In the study, we investigated the pharmacological mechanisms.

**Methods:**

The main components of QWS aqueous extract were analyzed by LC–MS, and potential pathways of QWS targeting OU were predicted by network pharmacology. The db/db mice were administered with the decoction of dried *Zingiber officinale* Rosc. rhizome combined with NaOH cauterization to establish the model of diabetic OU subjected to stomach heat syndrome. Subsequently, the model mice were treated with QWS, and OU wound healing status were recorded. The pathological changes of gastric tissue and oral mucosa were evaluated using hematoxylin–eosin staining, and the morphology of collagen fibers in oral mucosa was assessed by Masson staining. The levels of thromboxane B_2_ (TXB_2_), 6-Keto-prostaglandin F1α (6-keto-PGF1α), interleukin-1 β (IL-1β), IL-2, IL-6, tumor necrosis factor-α (TNF-α), β-endorphin (β-EP) and 5-Hydroxytryptamine (5-HT) were determined by ELISA assay. The protein expressions of Toll-like receptor 4 (TLR4), TNF receptor associated factor 6 (TRAF6), myeloid differentiation factor 88 (MyD88), inhibitor of NF-κB alpha (IκΒα), p-IκΒα and nuclear factor kappa-B (NF-κB) p65 were measured by Western Blotting.

**Results:**

A total of 183 compounds in QWS were identified by LC–MS, and identified 79 bioactive compounds corresponded to 269 targets and 59 pathways. QWS high-dose treatment significantly reduced the level of TXB_2_ and the ratio of TXB_2_/6-keto-PGF1α. Meanwhile, it improved mucosal pathological morphology, and reduced the area of OU and local edema. Simultaneously, the levels of TNF-α, IL-1β, IL-6, IL-2 and 5-HT, and the expressions of TLR4, TRAF6, MyD88, p-IκΒα and NF-κB p65 were decreased.

**Conclusion:**

QWS treatment facilitates the healing of OU, ameliorates pathological morphologies of gastric and oral mucosa and decreases the levels of pro-inflammatory cytokines in db/db mice subjected to stomach heat syndrome, whose mechanism may be associated with the inhibition of TLR4/MyD88/NF-κB signaling pathway to exert anti-inflammatory effects.

**Supplementary Information:**

The online version contains supplementary material available at 10.1186/s13020-021-00565-5.

## Background

Oral ulcer (OU) is a common oral mucosal disease, which mainly manifested by isolated or multiple, symmetric or random shaped ulceration of mucosa, and is characterized by small and round yellow or gray ulceration based erythema-like edge accompanied by intensive pain [1, 2]. Many kinds of acute OU belong to self-limiting diseases; however, such lesions can be recurrent with long recovery time, and also arise secondary to immune disorder, infectious and many physiopathological causes [3], leading to inconvenience and pain to patients. OU is also one of the complications in diabetes mellitus (DM), and about 45% patients with DM are suffered from it, whose pathogens are mainly attributed to fungal infection [4]. Diabetes-induced hyperglycemia results in the difficult-to-heal chronic wounds due to the upregulation of pro-inflammatory cytokines, damage of newly formed vessels, decreased collagen synthesis, elevated protease, and functional disorder of macrophage. Eventually, it results in recurrent oral mucosal lesion, further prolongs recovery time and aggravates OU [5–7].

In traditional Chinese Medicine (CM), the name of OU is recorded in *Huangdi’s Internal Classic-SuWen* (黄帝内经·素问) for the first time [8]. The pathological theory of CM on OU is attributed to irregular dietary habits resulting in dysfunction of stomach, which produces stomach heat along stomach meridian up to oral cavity to cauterize oral mucosa [9, 10]. DM usually belongs to the scope of *Xiaoke* (消渴) in CM, and stomach heat is one of primary pathologies for DM. Therefore OU is a common complication due to Qi-blood stagnation and injury of vessels by stomach heat [11]. QWS is developed by *Li Gao* (李杲) in his monograph *Secret Record of the Orchid Chamber* (兰室秘藏) in Jin dynasty, and the classic prescription is composed of *Rhizoma Cimicifugae* (Sheng Ma), *Rhizoma Coptidis* (Huang Lian), *Radix Angelicae Sinensis* (Dang Gui), *Radix Rehmanniae Recens* (Sheng Di Huang) and *Cortex Moutan* (Mu Dan Pi). It has been widely used in clinic for efficacies of clearing stomach heat and cooling blood, especially is suitable for treating OU, periodontitis and halitosis subjected to stomach heat syndrome [12]. However, few researches have focused on QWS intervening in diabetic OU presently, and ambiguous therapeutic effects and pharmacological mechanisms hinder wider application of QWS in clinical practice. In this study, we established a pathological model of OU subjected to stomach heat syndrome in db/db mice, and evaluated pharmacological efficacy of QWS on OU. By using LC–MS and network pharmacology analysis, we figured out active compounds of QWS corresponding to potential targets and pathways and further discovered the mechanisms were related to TLR4/MyD88/NF-κB signaling pathway. Our study provides scientific evidences for QWS in treating diabetic OU subjected to stomach heat syndrome by application with modern pharmacological technologies.

## Materials and methods

### Preparation of QWS and dosage conversion between human and mice

*Rhizoma Cimicifugae* (Sheng Ma), *Rhizoma Coptidis* (Huang Lian), *Radix Angelicae Sinensis* (Dang Gui), *Radix Rehmanniae Recens* (Sheng Di Huang), *Cortex Moutan* (Mu Dan Pi) and dried *Zingiber officinale* (Gan Jiang) were purchased from Beijing Taiyang Shukang Pharmaceutical Co. Ltd., China. QWS is an ancient classic prescription with variable doses from ancient to modern clinic. In order to investigate its therapeutic effect, we converted the dose in the monograph *Secret Record of the Orchid Chamber* and modern clinical dose into animal’s doses, respectively. According to ancient dosage standard of Chinese herbal medicine: 1 Qian = 3.72 g [13], five Chinese herbal medicines of QWS doses in monograph *Secret Record of the Orchid Chamber* were converted into Sheng Ma 3.7 g, Huang Lian 1.2 g, Dang Gui 1.2 g, Sheng Di Huang 1.2 g, Mu Dan Pi 1.9 g. According to the conversion ratio between mice and humans (10), the daily dose (g/kg) of QWS for mice can be calculated as the following formula: conversion ratio of surface area between mice and humans (10) × a daily dose of an adult (9.2 g)/average weight of adult (70 kg), that is: 10 × 9.2 (g)/70 (kg) = 1.32 g/kg. However, modern clinical dosages in the prescription are Sheng Di Huang 6 g, Dang Gui 6 g, Mu Dan Pi 9 g, Huang Lian 6 g and Sheng Ma 9 g, and the equivalent dose of mice is 5.14 g/kg. Therefore, in the study, 1.32 g/kg is regarded as low dose and 5.14 g/kg is as high dose.

According to the dose ratio of primary prescription, five herbal medicines were pulverized and passed through a 10-mesh sieve. The powder was weighed and placed in a ceramic pot, eightfold volume of water was added and decocted for 30 min. The aqueous extract was filtrated using a double-layer of gauze, and the residue was re-suspended in an eightfold volume of water and re-decocted for 30 min. The filtrated decoctions from the first and second times were mixed, subsequently concentrated to the desired concentration (13.2 g crude drug/L or 51.4 g crude drug/L) by simmering, consequently was regarded as QWS aqueous extract. The decoction of dried *Zingiber officinale* Rosc. rhizome (1 kg crude drug/L) was prepared according to the above method.

### LC–MS analysis and identification for compounds of QWS

QWS aqueous extract was centrifuged at 15,000 r/min for 10 min, and the supernatant was collected. After filtering through 0.22 μm filter membrane, filtrated fluid was used as test solution. Chromatographic separation was performed with a Vanquish UHPLC system (Thermo Fisher Scientific, Germany). EclipsePlus C18 RRHP (2.1 mm × 100 mm, 1.8 μm, Agilent Technologies, USA) column was applied with a constant flow rate of 0.3 mL/min at 35 ℃, and injection volume was 10 μL. The mobile phase consisted of 5 mmol/L ammonium acetate aqueous solution containing 0.1% formic acid (A) and methanol (B), using a gradient elution of 5% B at 0–5 min, 5–80% B at 5.1–15 min, 80–0% B at 15.1–17 min, 0% B at 17.1–20 min. Mass spectrometric analysis was performed using Q-Exactive HF high-resolution mass spectrometry (Thermo Fisher Scientific, Germany). An electrospray ionization source (ESI) was operated in positive and negative ion modes, respectively. Scan mode: Full-MS/dd-MS^2^ with a resolution of 120,000/60000; scan range: m/z 100–1500; ion spray voltage: 3500 V; capillary temperature: 330 °C; sheath gas flow rate: 45 arb; auxiliary gas flow rate: 15 arb; curtain gas: 35 psi, and collision induced dissociation (CID) parameter was set as medium. Peak alignment, retention time correction, and peak area extraction were performed using Compound Discoverer 3.2. Preliminary identification of compounds was realized by searching databases including mzCloud (Thermo Fisher Scientific), mzVault, and local database.

### Construction of pharmacology network

The canonical SMILES of each identified compound was obtained from PubChem database (https://pubchem.ncbi.nlm.nih.gov/) and then uploaded into SwissADME database [14] (http://www.swissadme.ch/index.php) to evaluate gastrointestinal absorption (GA) and drug-likeness (DL). If the compound showed “high” in GA and met more than two of the five filters (Lipinski, Ghose, Veber, Egan, Muegge) in DL, it would be a candidate compound [15]. Then the canonical SMILES of candidate compound was imported into SwissTargetPrediction database [16] (http://www.swisstargetprediction.ch/) for target prediction, “Homo sapiens” was selected as species, and targets with probability > 0 were included. OU related targets were obtained from DisGeNET database [17] (https://www.disgenet.org/) and GeneCards database [18] (https://www.genecards.org/), and standardized using UniProt database [19] (https://www.uniprot.org/). The overlapping targets between candidate compounds targets and OU related targets were considered as potential therapeutic targets. The overlapping targets were used to construct protein–protein interaction (PPI) network by STRING database [20] (http://string-db.org/). The research species was defined as “Homo sapiens”, and the minimum required interaction score was set to 0.9. The potential therapeutic targets were imported into DAVID database [21, 22] (https://david.ncifcrf.gov/tools.jsp) for gene ontology (GO) and Kyoto Encyclopedia of Gene and Genome (KEGG) pathway enrichment analysis. The compounds-targets-pathways interaction network was constructed using Cytoscape3.7.2 [23] to provide information about the interactions among active compounds, targets and pathways.

### Establishing OU model subjected to stomach heat syndrome in db/db mice

Male db/db mice (C57BLKS/J Leprdb/db, 6 weeks, 40 g average body weight) and control lean nondiabetic mice (C57BLKS/J lar-m +/m +, male, 6 weeks, 20 g average body weight) were purchased from Chang Zhou Cavens Laboratory Animal Ltd. The experimental animal production license number is SCXK (Su) 2018-0002. All mice were fed in barrier environment of Beijing University of Chinese Medicine, and the experimental animal use license number is SYXK (Jing) 2020-0033. Animals were housed under temperature at 22–24 °C, relative humidity at 50–70%, and a 12 h light–dark cycle. All mice were allowed to acclimate to the environment for a week before the experiment. The animal protocol of this study was approved by Medical and Experimental Animal Ethics Committee of Beijing University of Chinese Medicine (No. BUCM-4-2021030504-1036) and Animal Research Ethics Sub-Panel of University of Macau (No. UMARE-008-2021).

Experiment was started on day 0 (D0). The db/db mice with random blood glucose levels ≥ 11.1 mmol/L were administered by gavage with the decoction of dried *Zingiber officinale* Rosc. rhizome at a dose of 8 g/kg for 14 days (D1 to D14) to establish T2DM subjected to stomach heat syndrome model (T2DM + SH) [24]. The normal control group (m/m mice) and T2DM group (db/db mice) were administered an equal volume of distilled water. On the 15th day (D15), mice in T2DM + SH and T2DM groups were anesthetized with 2% isofluorane by inhalation. To establish T2DM + SH + OU and T2DM + OU models, NaOH crystals (045780, Beijing inokai Technology, China) with a diameter of about 2 mm were placed at the left buccal mucosa for 10 s until mucosal ulcer or slight bleeding [25], and mice in the normal control group without any intervention. The location, depth, and size of OU in each mouse should be as consistent as possible. The formation of ulcers after 24 h was observed and two T2DM + SH + OU mice were randomly selected and sacrificed. The excised tissues from OU mucosa and stomach were performed with 4% formaldehyde fixed, paraffin embedded and section slice. Hematoxylin–eosin (HE) staining for the morphology of tissues in mice was observed under microscope.

### Group division and treatment

T2DM + SH + OU mice were randomly divided into four groups as follows (*n* = 14/group): T2DM + SH + OU group, the positive drug Kou Qiang Kui Yang San (口腔溃疡散, KQKYS) (20100003, Tong Ren Tang Pharmaceutical Factory, China) group, Qingwei San high dose (QWS-H) (5.14 g/kg) and low dose (QWS-L) (1.32 g/kg) group. In QWS groups, mice were administered with two corresponding concentrations of QWS by oral gavage, respectively, once a day; and in KQKYS group, KQKYS were smeared on the ulcer of mice, twice a day for 7 days (D16 to D22). Additionally, in the normal control, T2DM + SH + OU and T2DM + OU groups, mice were administered with an equal volume of distilled water by gavage once a day.

The mice in each group, including their activity, hair luster, water and food intake, body weight and fasting blood glucose level, were observed and recorded on D0/7/14/22.

### Ulcer healing assessment

During the procedure of treatment, the status of ulcers was observed and recorded, and the area of ulcers was quantified by Image J software.

$$ {\text{Wound healing rate}} = \left( {{\text{initial wound area}} - {\text{wound area at the time point}}} \right)/{\text{initial wound area }} \times \, 100\% $$

### Histopathological examination

After 7 days of QWS administration, the gastric and OU mucosal tissues of mice in each group were excised, and fixed with 4% paraformaldehyde for 48 h, dehydrated with ethanol (100092683, Sinopharm Chemical Reagent, China), transparentized with xylene (10023418, Sinopharm Chemical Reagent, China), embedded in paraffin, and cut into slices. HE staining (G1003, Servicebio, China) was used to observe the pathological morphology changes, and Masson staining (G1006, Servicebio, China) was applied to detect the collagen fiber of oral mucosa.

### ELISA assay

Concentrations of thromboxane B_2_ (TXB_2_) (KT2519-A, Jiangsu Kete biology, China) and 6-Keto-prostaglandin F1α (6-keto-PGF1α) (KT2264-A, Jiangsu Kete biology, China) in stomach tissue were determined using enzyme-linked immunosorbent assay (ELISA) kits. The blood of mice was obtained by eyeball extraction and the serum was separated by centrifugation. The serum levels of interleukin-1β (IL-1β) (KT2040-A, Jiangsu Kete biology, China), IL-2 (KT2698-A, Jiangsu Kete biology, China), IL-6 (KT2163-A, Jiangsu Kete biology, China), tumor necrosis factor-α (TNF-α) (KT2132-A, Jiangsu Kete biology, China), β-endorphin (β-EP) (KT2557-A, Jiangsu Kete biology, China) and 5-Hydroxytryptamine (5-HT) (KT2443-A, Jiangsu Kete biology, China) were detected using their respective ELISA kits. The specific operation was performed following kit’s instruction.

### Western blotting

The protein expressions of Toll-like receptor 4 (TLR4), TNF receptor associated factor 6 (TRAF6), myeloid differentiation factor 88 (MyD88), inhibitor of NF-κB alpha (IκΒα), p-IκΒα and nuclear factor kappa-B (NF-κB) p65 were measured with Western blotting. Oral mucosa tissue was lysed in RIPA Lysis Buffer containing protease and phosphatase inhibitors. BCA protein detection kit (P0010S, Beyotime, China) was used to detect protein concentration. The total protein (10 μg) of each sample was added to SDS-PAGE gel electrophoresis to separate the proteins and transferred to PVDF membrane. PVDF membrane containing protein was incubated in a closed solution for 2 h. PVDF membrane was incubated in the required primary antibodies, including TLR4 antibody (1:500, ab13556, Abcam, UK), MyD88 antibody (1:1000, ab219413, Abcam, UK), TRAF6 (1:5000, ab33915, Abcam, UK), IκBα antibody (1:2000, ab76429, Abcam, UK), p-IκBα antibody (1:1000, 2859S, CST, USA), NF-κB p65 antibody (1:2000, ab16502, Abcam, UK) and β-tubulin antibody (1:2000, 10094-1-AP, Proteintech, USA) overnight at 4 °C. After incubating with HRP-conjugated Affinipure Goat Anti-Rabbit IgG (H + L) (1:5000, SA00001-2, Proteintech, USA) for 2 h, PVDF membrane was washed with solution TBST, treated with chemiluminescence reagent, and exposed and photographed. Western blot bands were quantified using Image-Pro-Plus 6.0, and the relative expression levels were analyzed as the ratio of corresponding bands to β-tubulin.

### Statistical analysis

All data in this study were statistically analyzed using SPSS 22.0 and GraphPad Prism 8 software and expressed as mean ± standard deviation (SD) values. Statistical analysis was performed using one-way ANOVA (LSD *t*-test) and Student’s *t*-test. *P*-value < 0.05 was considered as statistical significance.

## Results

### Identification of chemical compounds from QWS aqueous extract

**Fig. 1:**
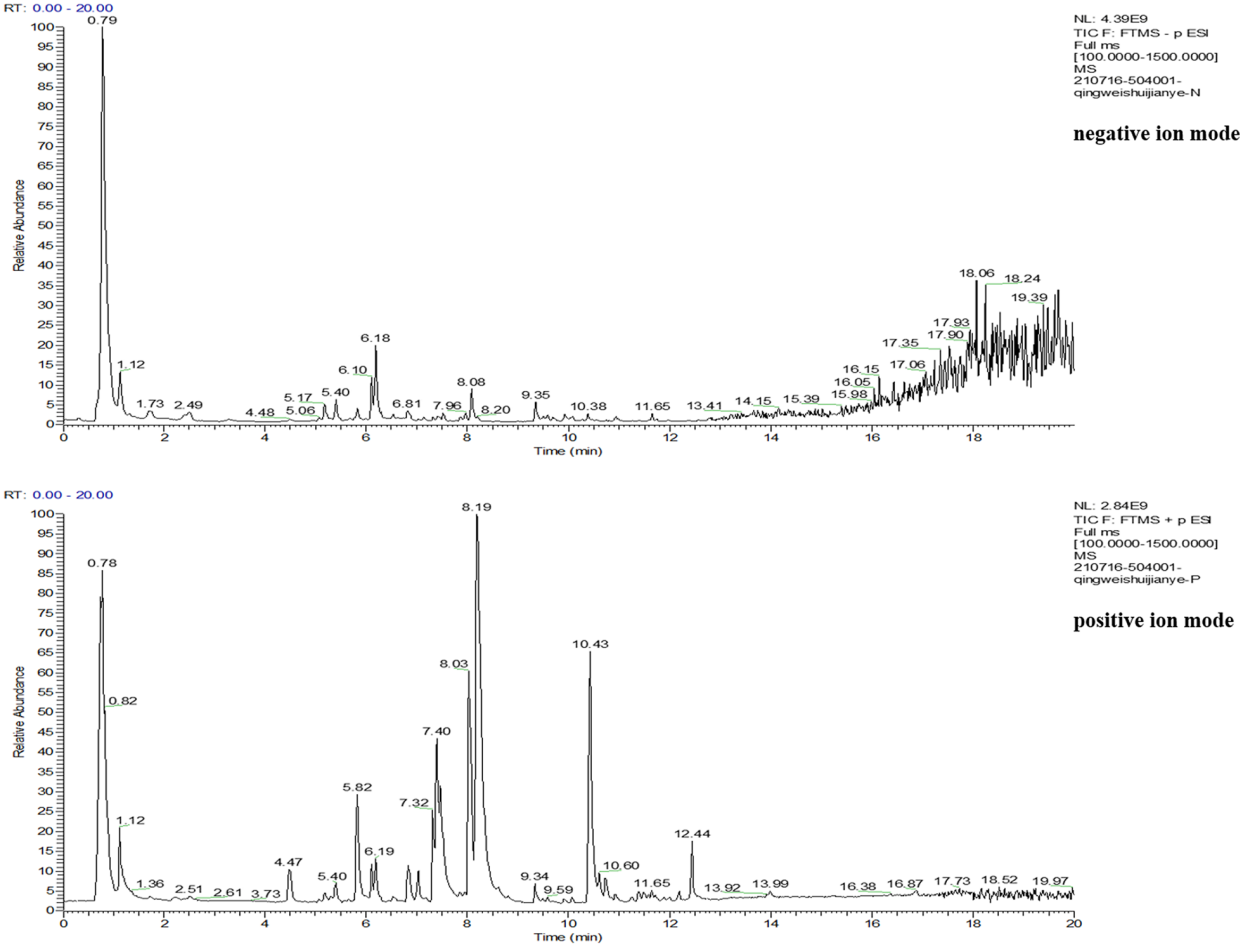
Total ion chromatogram of QWS aqueous extract

As shown in Fig. 1, the result of total ion chromatogram of mass spectroscopy illustrated that 183 compounds in QWS aqueous extract were identified through chromatogram matching. Additional file 1: Table S1 shows retention time, experimental and calculated molecular weight, molecular formulas, errors in parts per million (ppm), and major MS/MS fragments, etc. The mass error of all identified compounds was less than 5 ppm.

### Prediction of targets and pathways by network pharmacology technology

**Fig. 2:**
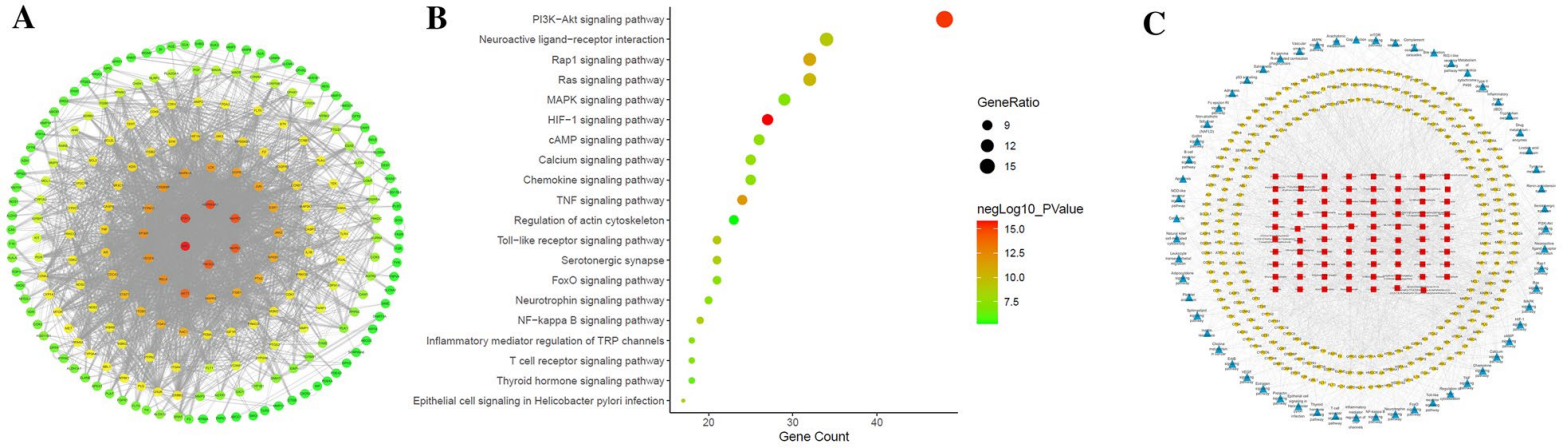
Network pharmacology analysis for screening targets and pathways of QWS. **A** PPI network of common targets. The color of node indicated the size of degree value; The greater the degree value corresponding to node color from green to red; The thickness of edge and combine score value have a positive correlation. **B** KEGG pathway analysis for common targets. **C** Component-target-pathway network

Firstly, 98 compounds were screened out by SwissADME based on DL and GA, and a total of 725 targets were obtained from these compounds, and 1356 OU related targets were gathered in corresponding databases after removing duplicate values. Then, a total of 269 overlapping genes were screened out through Venn analysis to be potential targets related to OU (Additional file 2: Fig. S1A), which corresponded to 79 of 98 compounds of QWS. PPI analysis was performed on above 269 targets through STRING database and used to build network further by Cytoscape software (Fig. 2A). The PPI network consisted of 218 nodes and 2241 edges and 30 nodes with degree value ≥ 11, betweenness centrality ≥ 0.01045, closeness centrality ≥ 0.3412 and average shortest path length ≤ 3.1944 were selected as major genes, including SRC, STAT3, HSP90AA1, MAPK3, PIK3CA, MAPK1, AKT1, RELA, EP300 and VEGFA. These genes were likely to play an important pharmacological role in OU process.

To further investigate the mechanisms of QWS on OU at a systematic level, 269 overlapping targets were uploaded into DAVID. The results of GO analysis showed that 246 biological processes (BP), 34 cell components (CC), and 57 molecular functions (MF) enriched for these targets were recognized (*P* < 0.01, FDR < 0.01). Top 20 terms in BP, CC and MF were presented in Fig.S1B-D based on gene count. Enriched BPs included signal transduction, positive regulation of transcription from RNA polymerase II promoter, response to drug, positive regulation of cell proliferation, inflammatory response. Enriched CCs included plasma membrane, cytoplasm, cytosol, extracellular exosome, nucleoplasm. Enriched MFs included protein binding, ATP binding, protein homodimerization activity, identical protein binding, enzyme binding. According to results of pathway enrichment, 59 target-related pathways had been found (*P* < 0.01, FDR < 0.01). Top 20 KEGG pathways were presented in Fig. 2B based on gene count. The KEGG enrichment analysis provided insight that QWS might act on PI3K-Akt signaling pathway, Neuroactive ligand-receptor interaction, Rap1 signaling pathway, Ras signaling pathway, MAPK signaling pathway, HIF-1 signaling pathway, TNF signaling pathway, Toll-like receptor signaling pathway, NF-kappa B signaling pathway.

In order to further explore the relationship between active ingredients of QWS and therapeutic effect on OU, a component-target-pathway network was constructed (Fig. 2C), which includes 79 compounds, 269 targets and 59 pathways. A total of 26 components (degree ≥ 16, betweenness centrality ≥ 0.01149, closeness centrality ≥ 0.3187 and average shortest path length ≤ 3.2080) were screened out as vital pharmacological chemicals of QWS on OU, such as 13(S)-HOTrE, 13S-hydroxyoctadecadienoic acid, 3,4-Dimethoxycinnamic acid, Ferulaldehyde, 4-Methylumbelliferone, Ferulic acid, etc. Multiple targets were associated with multiple compounds, indicating that different components in QWS had synergistic effects in the therapeutic process. And the targets were distributed in different pathways and were coordinated with each other. The results revealed that QWS treatment may exert therapeutic effects on OU through multiple pathways and multiple targets.

### The changes of water and food intake, weight body and fasting blood glucose level in mice after QWS treatment

**Fig. 3:**
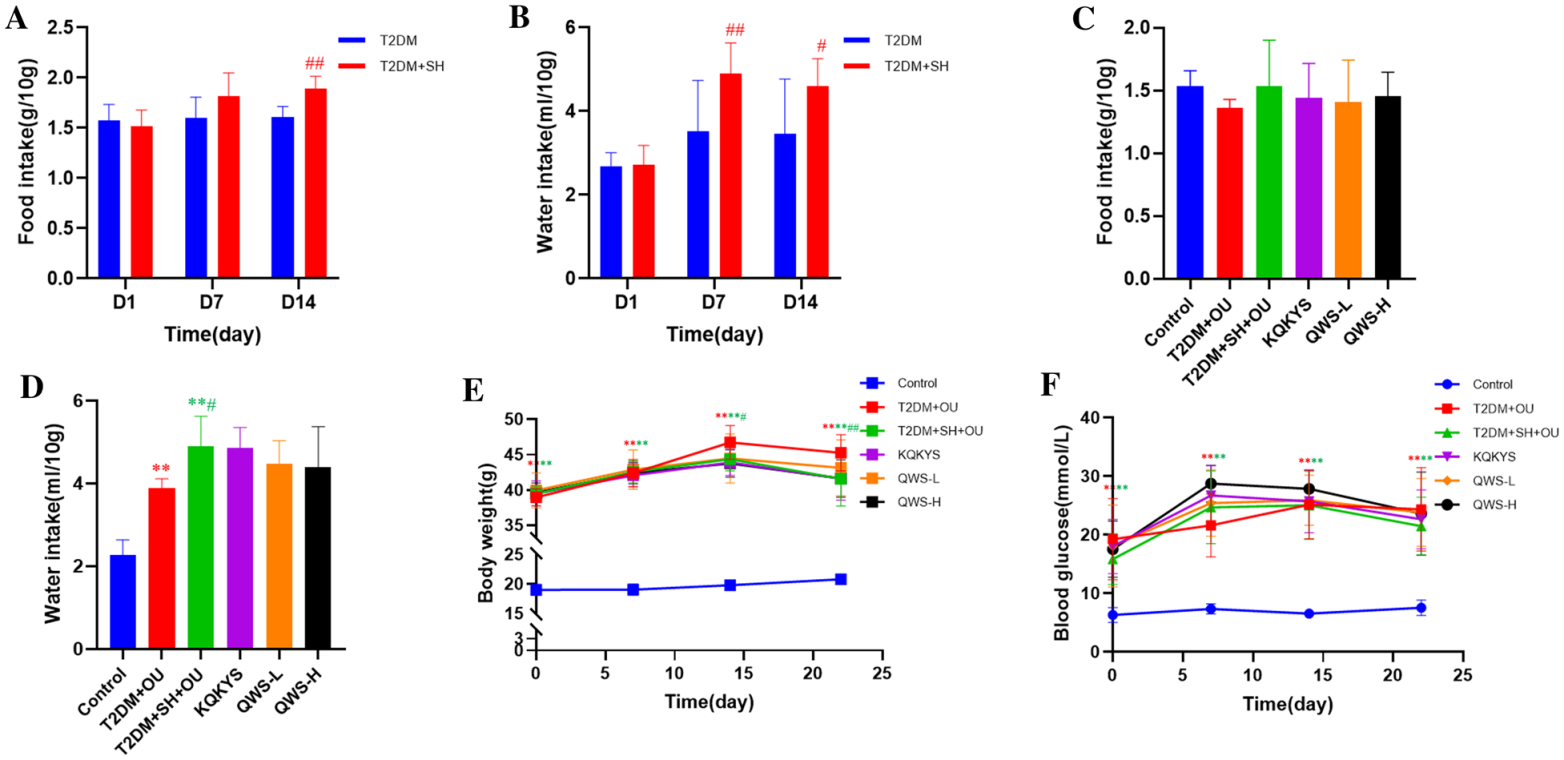
The changes of water and food intake, weight body and fasting blood glucose level in db/db mice subjected to stomach heat syndrome. **A** Comparison of food intake in mice between T2DM group and T2DM + SH group. **B** Comparison of water intake in mice between T2DM group and T2DM + SH group. **C** The changes of food intake after QWS treatment. **D** The changes of water intake after QWS treatment. **E** The changes of body weight after QWS treatment. **F** The changes of fasting blood glucose after QWS treatment. T2DM, type 2 diabetes mellitus; OU, oral ulcer; SH, stomach heat syndrome; QWS-L, Qingwei San aqueous extract (1.32 g/kg); QWS-H, Qingwei San aqueous extract (5.14 g/kg). Data were shown as mean ± SD. *P < 0.05, **P < 0.01 vs. normal control group; ^#^*P* < 0.05, ^##^*P* < 0.01 vs. T2DM + OU group; ^&^*P* < 0.05, ^&&^*P* < 0.01 vs. T2DM + SH + OU group

The mice in normal control and T2DM groups exhibited shiny hair, a certain level of movement vigor, and regular dietary, stool and urine. The mice in T2DM + SH group showed lusterless hair and hoarseness. They were more irritable and aggressive. Their daily urine volumes were less and more yellow, and their stool presented dry and hard. Compared with T2DM group, water and food intake of mice were notably increased (*P* < 0.01, Fig. 3A, B) and body weight was significantly decreased (*P* < 0.05, Fig. 3E) in T2DM + SH group, which were consistent with common signs or symptoms of stomach heat syndrome in humans with T2DM [26].

After 7 days of QWS administration (D22), mice suffered with OU showed lower food intake than that of normal control group, which might be associated with ulcers pain. Mice in T2DM + SH + OU group exhibited significantly increased water intake (*P* < 0.05) and decreased body weight (*P* < 0.01), and food intake showed an upward trend, compared with T2DM+OU group. The amount of food and water intake in QWS-L and QWS-H groups still showed a downward trend, and body weight in QWS-L group suggested an upward trend compared with T2DM + SH + OU group (Fig. 3C–E). These results illustrated that QWS treatment was able to ameliorate some stomach heat symptoms. However, there was no obvious effect of QWS on fasting blood glucose level (Fig. 3F). Considering short administration time of QWS (7 days), whether it has a hypoglycemic efficacy remains to be further studied. Compared with normal control group, body weight and fasting blood glucose level of db/db mice were significantly increased (*P* < 0.01, Fig. 3E, F), which was consistent with symptoms of T2DM.

### The characteristics of gastric and oral mucosa in T2DM + SH + OU mice model

**Fig.4:**
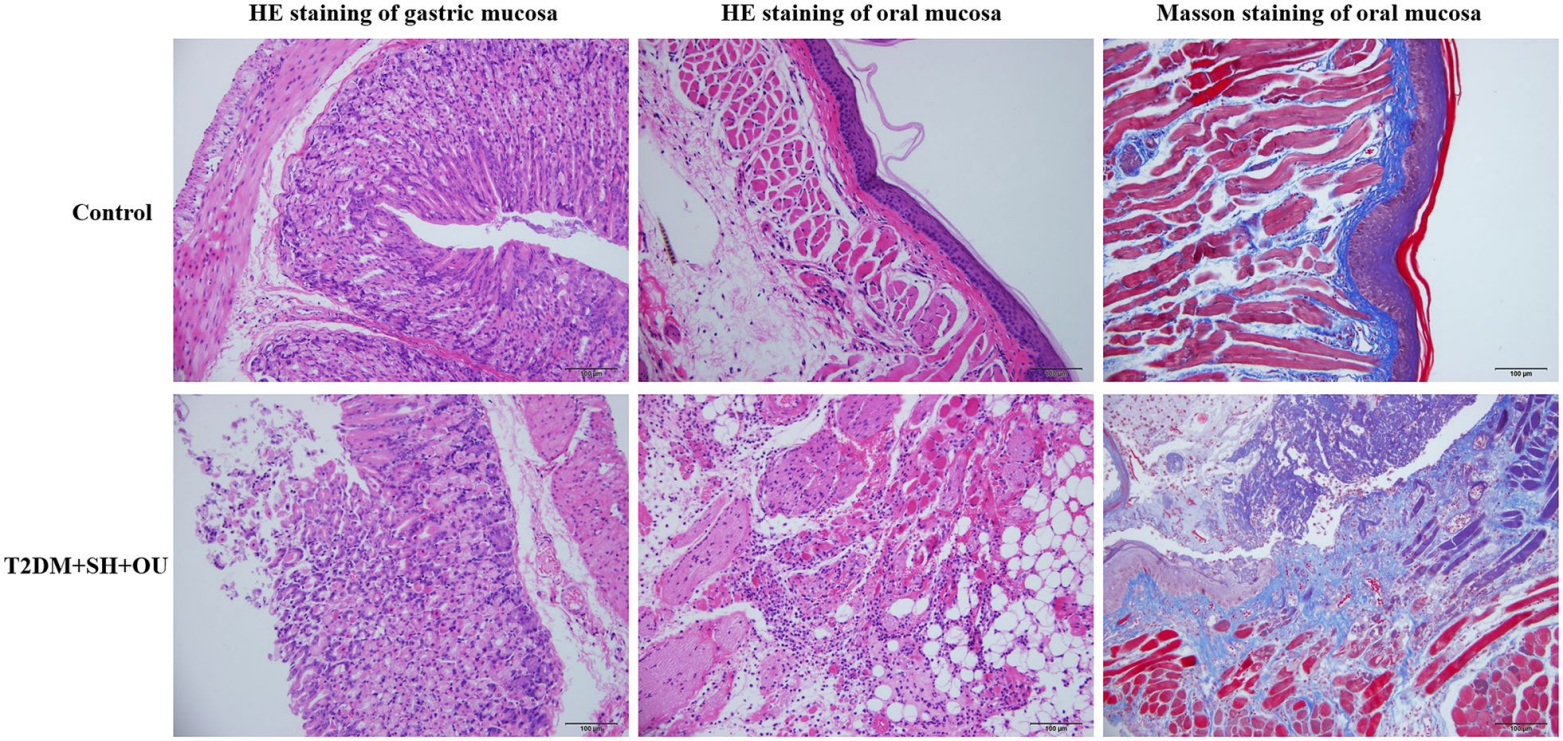
Histopathology of oral and gastric mucosa in stomach heat syndrome’s OU db/db mice (×200, scale bar = 100 μm)

Under observation of microscope, HE staining illustrated gastric mucosa edema and congestion, epithelial cell necrosis and shedding, accompanied by gland deformation and disordered arrangement in T2DM + SH + OU mice model, fulfilling the pathological criteria of stomach heat syndrome in humans [26] (Fig. 4). After inducing oral ulcer to db/db mice administered by the decoction of dried *Zingiber officinale* Rosc. rhizome for 24 h, a round-like ulcers appeared in left buccal mucosa. Ulcers surface was covered with gray-white or yellow pseudomembrane with a central depression and surrounding hyperemia edema, appearing obvious OU. Under observation of microscopy, oral mucosa exhibited severe injuries with massive infiltration of lymphocytes and neutrophils, and blood capillaries were dilated and congested, as well as collagen fibers were irregularly arranged, thin and broken (Fig. 4). It indicates that the establishment of stomach heat syndrome’s OU model in db/db mice was successful.

### QWS treatment ameliorated ulcer healing

**Fig.5:**
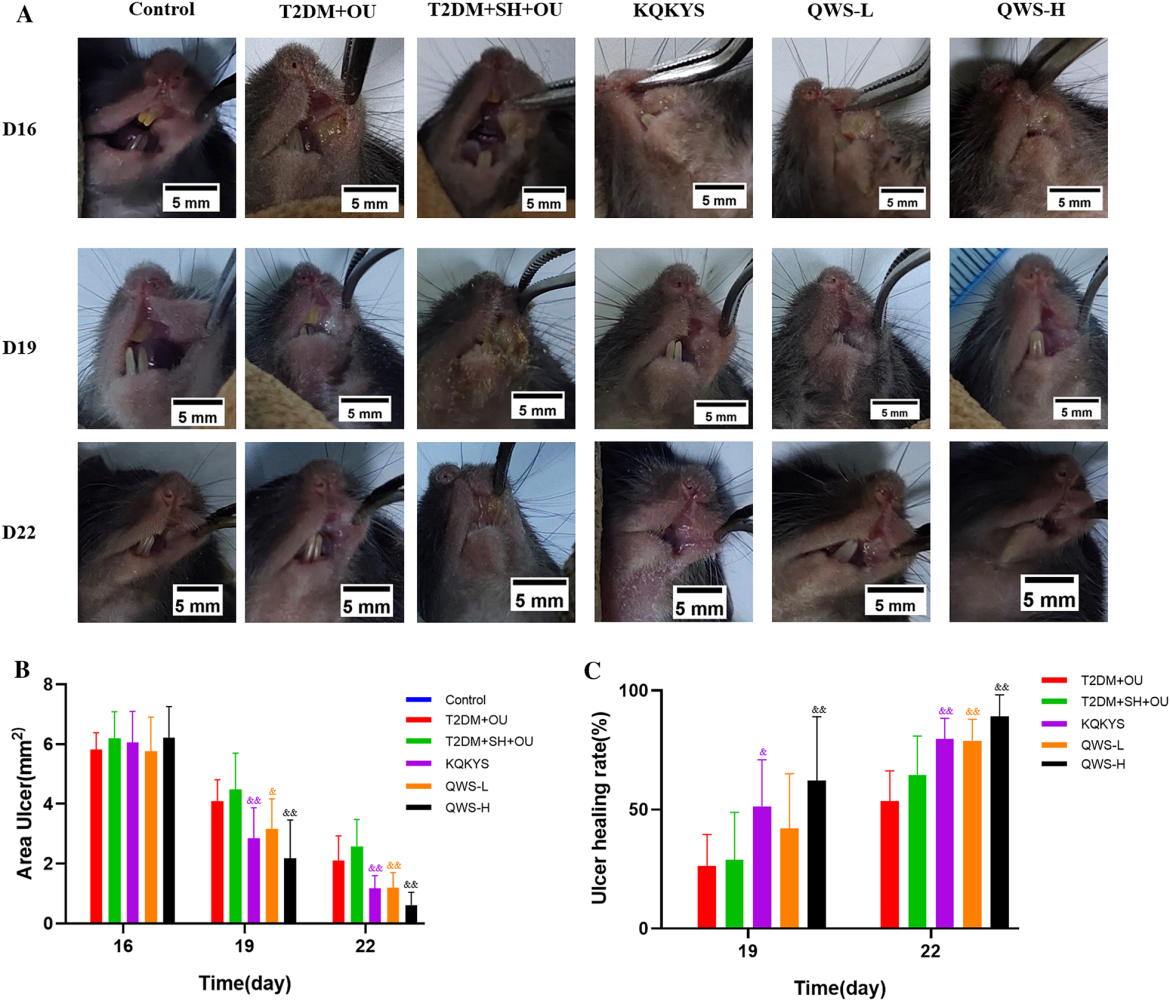
Therapeutic effect of QWS on ulcers healing. **A** Images of ulcers in six groups were presented. **B** Quantification of ulcers area. **C** Quantification of ulcer healing rate. T2DM, type 2 diabetes mellitus; OU, oral ulcer; SH, stomach heat syndrome; QWS-L, Qingwei San extract (1.32 g/kg); QWS-H, Qingwei San extract (5.14 g/kg). Data were shown as mean ± SD. ^&^*P* < 0.05, ^&&^*P* < 0.01 vs. T2DM + SH + OU group

As shown in Fig. 5A, in normal control group, oral mucosa of mice was pink, intact, smooth and no ulceration. While in other groups, it showed an obvious round-like OU on left cheek after cauterized by NaOH for 24 h, and no difference was observed in ulcers area among groups (*P* > 0.05). After low and high doses of QWS administration for four days (D19), ulcer area of mice was decreased in varying degrees, and ulcers became shallower with less congestion and edema. After seven days of treatment (D22), OU of mice in T2DM + SH + OU group and T2DM + OU group were still visible and mucosal hyperemia and edema around ulcer were decreased, and parts of pseudomembrane fell off from surface, but in QWS-treated mice, mucosa in injured area was similar to normal mucosa without obvious hyperemia and edema. No significant differences in ulcers area and wound healing rate were found between T2DM + OU group and T2DM + SH + OU group at D19 and D22 (*P* > 0.05). Compared with T2DM + SH + OU group at the same time point, ulcers area in QWS-L and QWS-H groups was notably reduced, and wound healing rate was definitely increased (*P* < 0.01). Although there was no significant difference in ulcers healing between KQKYS group and QWS-H group, high dose of QWS treatment showed better therapeutic effects on reducing ulcers area and improving ulcers healing rate (Fig. 5B, C).

### QWS treatment improved pathological morphology of gastric and oral mucosa

**Fig. 6:**
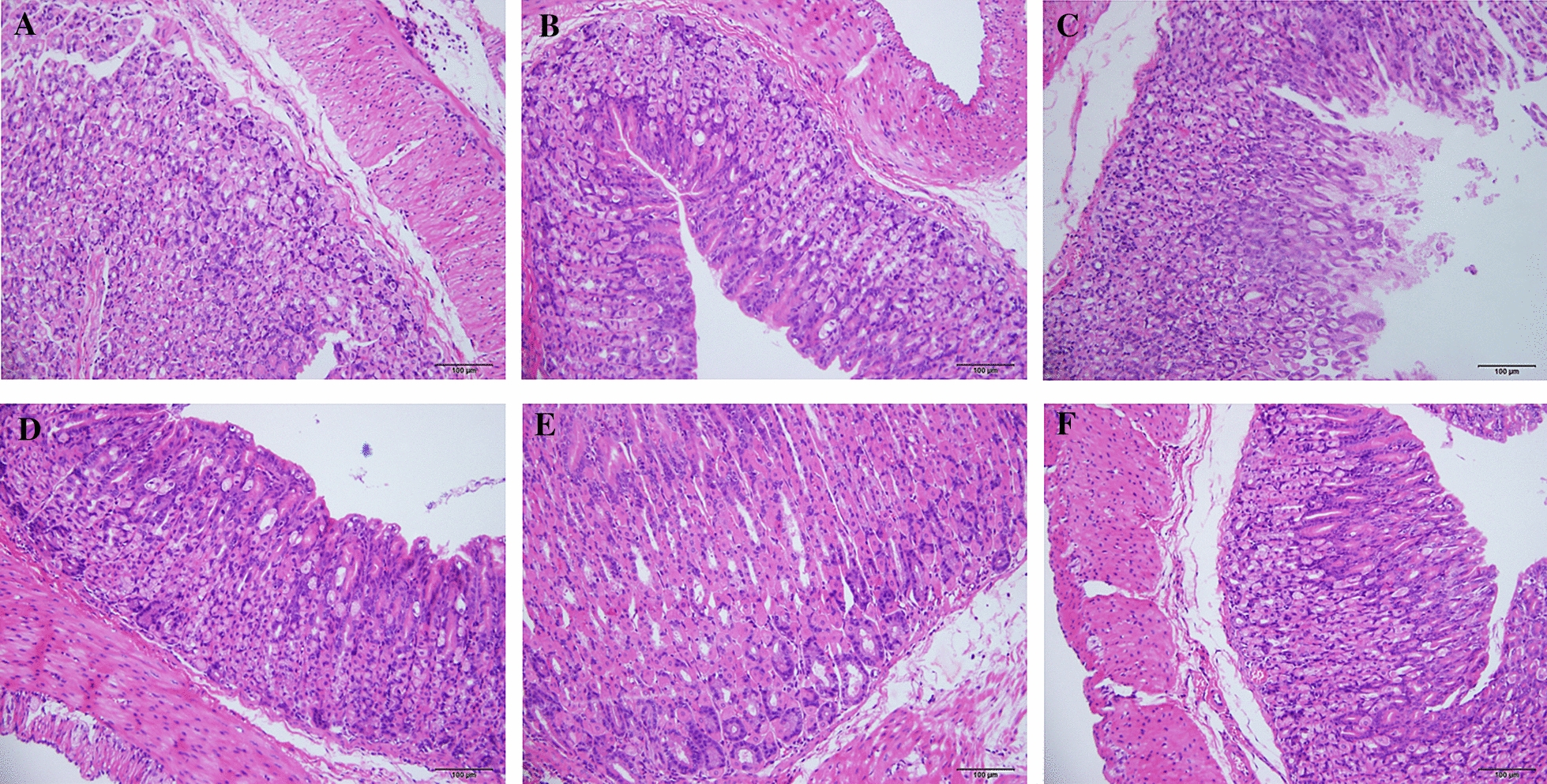
Histopathological examination of gastric mucosa. **A** normal control, **B** T2DM + OU, **C** T2DM + SH + OU, **D** KQKYS, **E** QWS-L, **F** QWS-H (HE, ×200, scale bar = 100 μm)

Gastric wall of mice in normal control and T2DM + OU groups showed distinct structure, and glands of lamina propria were clear, well-arranged, neither atrophy, shedding nor defect was observed. Gastric mucosa of T2DM + SH + OU mice was mildly damaged and a small number of epithelial cells shed off. Gastric mucosal structure of mice in QWS-L and QWS-H groups were intact, and epithelial cells and glands were arranged neatly, and mucosal hyperemia was obviously attenuated (Fig. 6).

**Fig. 7:**
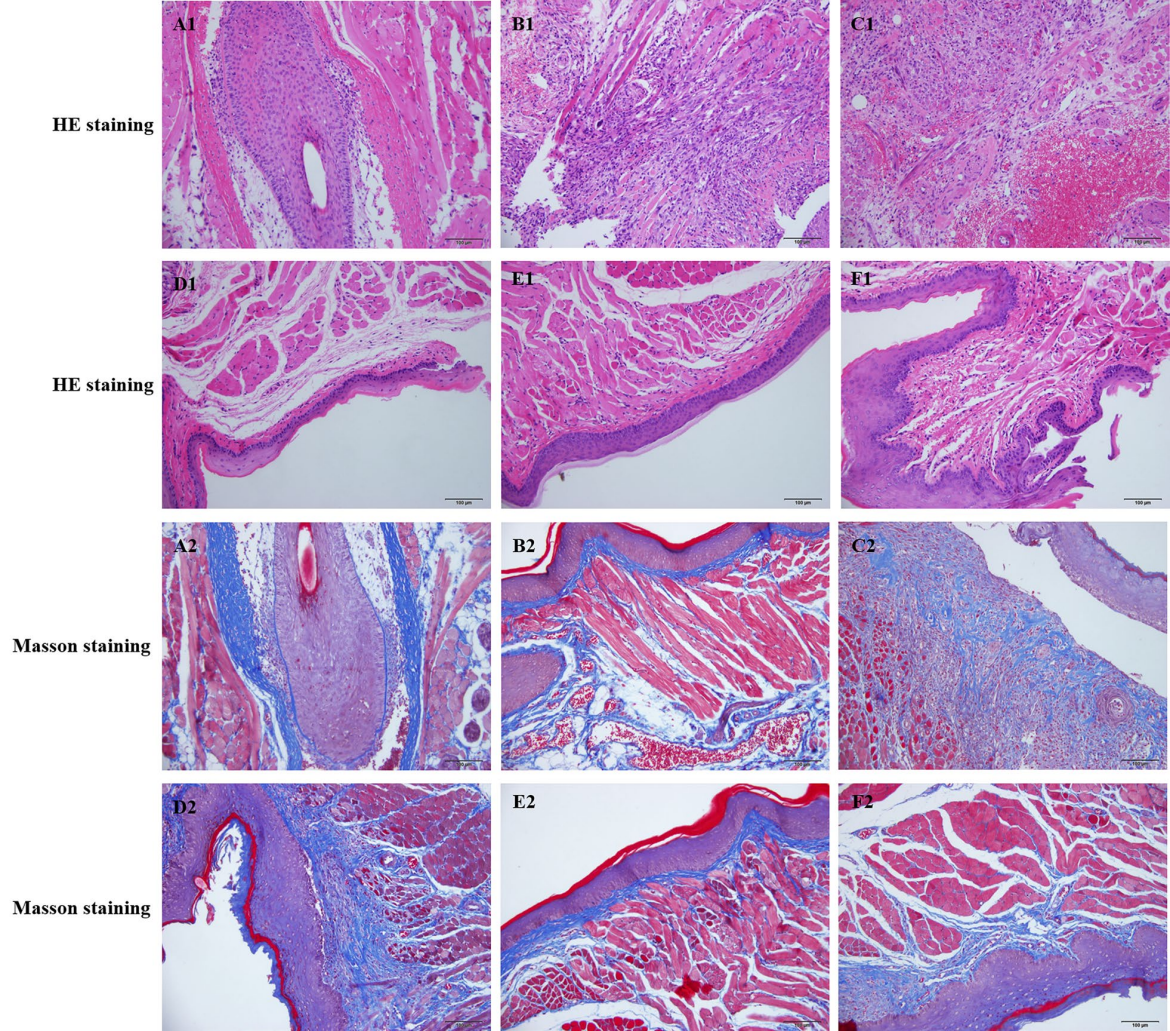
Histopathological examination of oral mucosa. **A** normal control, **B** T2DM + OU, **C** T2DM + SH + OU, **D** KQKYS, **E** QWS-L, **F** QWS-H (×200, scale bar = 100 μm)

In normal control group, oral mucosa tissue structure was distinct, and cell structure was intact with neatly arranged cells without inflammatory cells infiltration. In T2DM + SH + OU group, the integrity of oral mucosa was broken including shed epithelial cells and irregular and pyknotic nuclei and hyperchromatic change, and its surface was covered with necrotic tissue, as well as was infiltrated by a large number of neutrophils and lymphocytes, even presented focal pyogenic or hemorrhagic lesion in some cases. Histological lesions in oral mucosa were milder in T2DM + OU group than those in T2DM + SH + OU group. Pathologic degree of oral mucosa in QWS-L and QWS-H groups were significantly improved compared with that in T2DM + SH + OU group, and inflammatory cell infiltration was obviously relieved, with visible part of new epithelial proliferation and intact cell morphology (Fig. 7A1–F1).

Collagen fiber of oral mucosa was stained in blue. In normal control group, collagen fibers were abundant, evenly distributed and orderly. In T2DM + OU and T2DM + SH + OU groups, it showed broken, thin, disordered and scattered collagen fiber bundles. In QWS-L and QWS-H groups, collagen fibers were thick and intact, uniformly colored, and arranged neatly and regularly (Fig. 7A2–F2).

### QWS treatment decreased the levels of TXB_2_, inflammatory cytokines and 5-HT

**Fig. 8:**
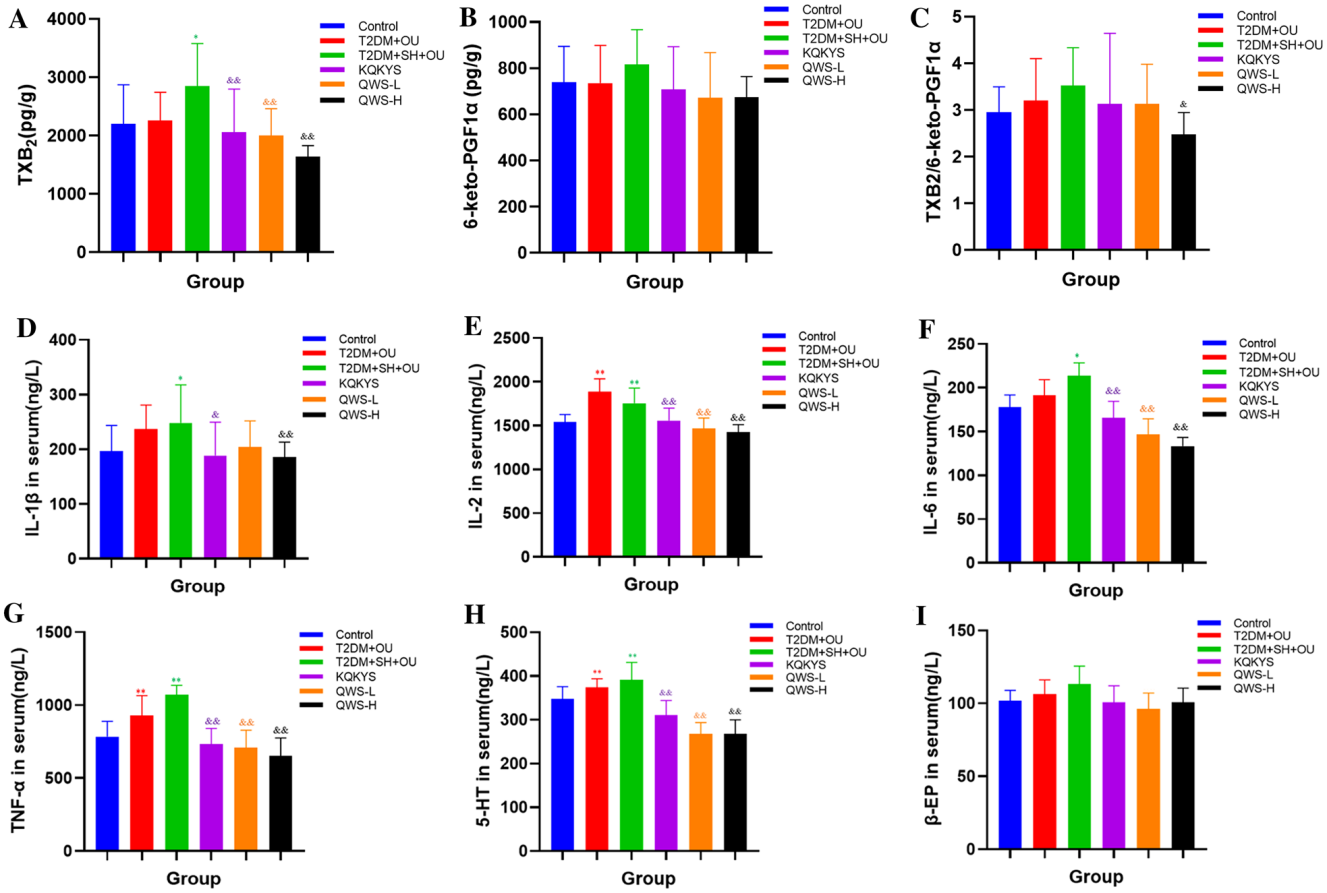
Effects of QWS on the levels of TXB_2_, 6-keto-PGF1α and inflammatory cytokines. **A** TXB_2_ level was quantified, **B** 6-keto-PGF1α level was quantified, **C.** the ratio of TXB_2_/6-keto-PGF1α was quantified. **D**–**G** the levels of IL-1β, IL-2, IL-6, TNF-α level were quantified. **H**, **I** 5-HT and β-EP levels were quantified. T2DM, type 2 diabetes mellitus; OU, oral ulcers; SH, stomach heat syndrome; QWS-H, Qingwei San extract (5.14 g/kg). Data were shown as mean ± SD (*n* = 10). *P < 0.05, **P < 0.01 vs. normal control group; ^&^*P* < 0.05, ^&&^*P* < 0.01 vs. T2DM + SH + OU group

No significant differences in the levels of TXB_2_ and 6-keto-PGF1α in gastric tissue were observed between normal control and T2DM + OU groups (*P* > 0.05). In comparison with normal control group, the levels of TXB_2_ and the ratio of TXB_2_/6-keto-PGF1α were increased in T2DM + SH + OU group, although there was no statistic difference. Compared with T2DM + SH + OU group, low and high doses of QWS and KQKYS treatments significantly reduced TXB_2_ level (*P* < 0.01), and high dose of QWS treatment markedly decreased the ratio of TXB_2_/6-keto-PGF1α (*P* < 0.05) (Fig. 8A–C).

Compared with normal control group, the levels of TNF-α and IL-2 in serum were increased significantly in T2DM + OU group (*P* < 0.01), and the levels of IL-1β, IL-2, IL-6 and TNF-α presented obviously high in T2DM + SH + OU group (*P* < 0.05). There were no significant differences in the levels of IL-1β, IL-2, IL-6 and TNF-α between T2DM + OU group and T2DM + SH + OU group, although the levels of IL-1β, IL-6 and TNF-α in T2DM + SH + OU group were higher than those in T2DM + OU group. Compared with T2DM + SH + OU group, low and high doses of QWS treatment significantly reduced IL-2, IL-6 and TNF-α levels (*P* < 0.01), and high dose of QWS treatment markedly decreased IL-1β level (*P* < 0.01). Although there was no significant difference in serum inflammatory factors between KQKYS and QWS-H groups, the suppression effect on inflammatory factors was more pronounced in QWS-H group (Fig. 8D–G).

Compared with serum 5-HT level in normal control group, it was significantly increased in T2DM + OU and T2DM + SH + OU groups (*P* < 0.01), while serum β-EP concentration showed no statistical significance. Additionally, there were no significant differences in the levels of 5-HT and β-EP between T2DM + OU and T2DM + SH + OU groups. Compared with T2DM + SH + OU group, treatments of QWS and KQKYS notably reduced 5-HT level, respectively (*P* < 0.01), and QWS treatment failed to influence β-EP level (*P* > 0.05) (Fig. 8H, I).

### QWS treatment downregulated TLR4/MyD88/NF-κB pathway

**Fig. 9:**
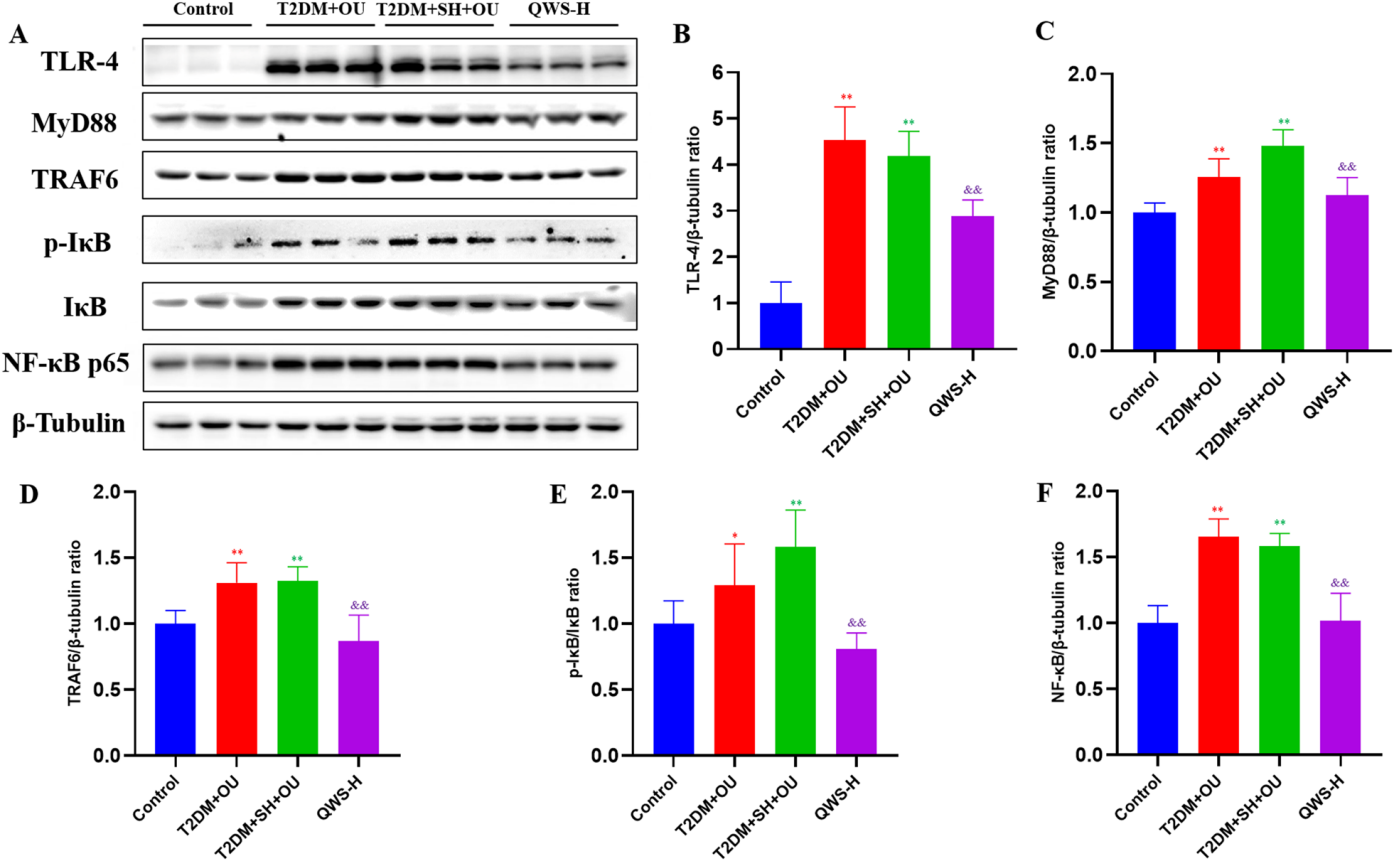
QWS treatment suppressed TLR4/MyD88/NF-κB pathway. **A** Western blotting assay was performed to measure the expressions of TLR4, MyD88, TRAF6, IκΒα, p-IκΒα and NF-κB p65 in oral mucosa. **B**–**F** Relative expressions of TLR4, MyD88, TRAF6, p-IκBα/IκBα and NF-κB p65 were quantified. T2DM, type 2 diabetes mellitus; OU, oral ulcers; SH, stomach heat syndrome; QWS-H, Qingwei San extract (5.14 g/kg). Data were shown as mean ± SD (*n* = 3). *P < 0.05, **P < 0.01 vs. normal control group; ^&^*P* < 0.05, ^&&^*P* < 0.01 vs. T2DM + SH + OU group

As shown in Fig. 9, the protein expressions of TLR4, TRAF6, MyD88, p-IκΒα, NF-κB p65 and the ratio of p-IκBα/IκBα in oral mucosa in T2DM + OU and T2DM + SH + OU groups were obviously increased compared with normal control group (*P* < 0.05). There were no marked differences of protein expressions mentioned above between T2DM + OU and T2DM + SH + OU groups. High dose of QWS treatment decreased the expressions of TLR4, TRAF6, MyD88, p-IκΒα and NF-κB p65, as well as downregulated the ratio of p-IκBα/IκBα compared with T2DM + SH + OU group (*P* < 0.01).

## Discussion

Diabetic OU is a kind of oral mucosa lesion which commonly occurs in lips, cheeks, tongue and palate. Traditional efficacy of QWS is clearing stomach heat [27]. In present study, we demonstrated that QWS could effectively treat OU subjected to stomach heat syndrome in db/db mice, and its potential mechanisms were related to inhibition of inflammatory injury by suppression of TLR4/MyD88/NF-κB signal pathway.

The LC–MS technique, with high separation properties of liquid chromatography and powerful identification ability of mass spectrometry, has been widely applied in separation and identification of complex components in TCM [28]. In this study, we established a reliable and effective LC–MS method, and 183 constituents, including 18 organooxygen compounds, 10 carboxylic acids and derivatives, 10 prenol lipids and other compounds, were identified in the chemical profile of QWS. Network pharmacology analysis can investigate the relationships among active compounds, target genes, and pathways, and establish a “compound-target-pathway” network to identify pharmacologically active compounds acting on specific targets and signaling pathways. In this study, 79 of the 183 identified compounds of QWS were considered to have potential therapeutic effect in OU by acting on 269 targets. The network topology parameter analysis results showed that 4-Methylumbelliferone, Ferulaldehyde, Ferulic acid, Ceratodictyol and Caffeic acid, etc. had high degrees, were more important compounds in QWS. Modern studies have also suggested pharmacological activities of these compounds, such as anti-inflammatory, analgesic, antioxidant, anti-tumor and antimicrobial effects [29–34]. The targets associated with multiple compounds in the network also reflected synergistic effects between different compounds during therapeutic process.

In this experiment, db/db mice with stomach heat syndrome exhibited abnormal physiological characteristics with dried and small granular feces, yellow urine and light body weight while increased food and water intake, and pathological morphology changes and ulcers also appeared in gastric mucosa, suggesting successful establishment of T2DM + SH + OU model [35]. After QWS treatment, pathologic abnormalities of gastric mucosa were significantly recovered, demonstrating excellent efficacy of QWS on treating stomach heat syndrome in OU db/db mice. Thromboxane A2 (TXA_2_) is a type of thromboxane synthesized and released by activated platelet, whose effects have a strong promoting vasoconstriction and platelet aggregation resulting in spasm of small arteries in gastric submucosa and capillary thrombosis, eventually leading to mucosal ischemia, hypoxia, cell degeneration and necrosis, as well as damage of mucosal barrier, H^+^ diffusion, formation of ulcers and hemorrhage [36]. The half-life of TXA_2_ is about 30 s, then it is metabolized to inactive and stable TXB_2_, so the level of TXB_2_ can represent the level of TXA_2_. Prostacyclin (PGI_2_) is a subtype of type 1 prostaglandin, which is the highest expressional level and strong biological activity in gastric mucosal tissue, and exerts multiple actions on gastrointestinal mucosal protection and ulcers healing by inhibiting gastric acid secretion, stimulating tissue repair process, enhancing gastric mucosal blood flow and maintaining mucosal integrity. The half-life of PGI_2_ is only 2–3 min and then transformed into a relatively stable metabolite 6-keto-PGF1α, so the level of 6-keto-PGF1α can represent the level of PGI_2_, indirectly. Under physiological conditions, the levels of TXA_2_ and PGI_2_ formed a relative balance to keep biological homeostasis. In our study, we found that db/db mice subjected to stomach heat syndrome showed significantly high TXB_2_ and the ratio of TXB_2_/6-keto-PGF1α levels in gastric tissue and were regarded as potential factors of gastric mucosal injury. QWS administration decreased the level of TXB_2_ and the ratio of TXB_2_/6-keto-PGF1α in high dosage group, and further ameliorated gastric mucosal pathological changes, suggesting its efficacy of clearing stomach heat.

Once OU occurs, the lesion area generates acute and intense pain with a large number of inflammatory cells infiltration under mucosa and epithelial cells are injured and shed [37], and the pathologic progression is divided into three important stages: inflammation, proliferation and remodeling [38]. In clinic, general principles for treating OU are concentrated anti-inflammation, analgesic, and facilitating healing [39]. In our study, we utilized NaOH cauterization to establish db/db mice OU model, and treated with QWS for 7 days. We observed that ulcer surface was almost healed with smooth mucosal tissue, and less inflammatory cells were infiltrated together with continuous and orderly arrangement of new collagen fibers in mucosal tissue. All of pathological improvements indicated that QWS administration exerted significant therapeutic effects on OU recovery in mice.

As an endogenous opioid peptide, the action of β-EP is similar to morphine, which plays a very important role in against body pain. β-EP can regulate substance P and neurotransmitters (e.g., gamma-Aminobutyric acid) through multiple pathways, leading to reduction of excitatory pain signals into central nervous system or enhancement of dopamine release, which contribute to analgesic effect. 5-HT is released by mast cells and platelets involved in pathophysiologic pain, when tissues are damaged or undergoing inflammation. Studies indicated that 5-HT stimulated nociceptors or transient receptor potential V1 (TRPV1) channel activation resulting in hyperalgesia [40, 41]. Our study demonstrated that serum 5-HT levels in T2DM + OU and T2DM + SH + OU groups were high, while after QWS treatment, it was decreased. Additionally, the β-EP level presented no significant difference in low–high dose of QWS group compared with T2DM + SH + OU group. Therefore, we hypothesize that QWS relieves OU pain mainly by regulation of 5-HT instead of β-EP.

Evidence suggests the production of cytokines, e.g. TNF-α, IL-6, IL-2, IL-1β account for the occurrence of OU [42–44]. TNF-α is a common pro-inflammatory cytokine mainly generated by activated T cells, as a very early intermedium response to mucosal injury. It can also cooperate with IL-1β to trigger inflammatory cascade reaction, which induces the productions of IL-6, IL-2 and other inflammatory factors, increases vascular endothelial cells permeability, proliferation, differentiation and migration of oral mucosal epithelial lymphocytes and further release of a large number of specific or non-specific inflammatory factors, consequently causes focal immune damage [45]. In addition, TNF-α inhibits the activation of transforming growth factor beta (TGF-β), thereby suppressing the proliferation and synthesis of collagen, and the increase of IL-6 has a negative impact on angiogenesis, leading to delayed wound healing [46, 47]. Our results indicated that QWS treatment significantly reduced the levels of serum IL-1β, IL-2, IL-6 and TNF-αin mice, indicating it contributes to anti-inflammation of OU and facilitation of wound healing.

To explore the mechanisms of QWS treatment for OU, we used network pharmacology technology to figure out the potential targets and pathways. The results demonstrated that 79 compounds in QWS were closely related to 269 targets, involving 59 signal pathways, including PI3K-Akt, Ras, MAPK, TNF, TLRs, NF-κB signal pathways and so on. TLRs are a type of mediator involved in body’s immune response, and can non-specifically bind to pathogen-related molecular patterns, initiate signal transduction, and ultimately lead to activation of NF-κB pathway, further trigger the release of inflammatory mediators. TLRs can be expressed in a variety of cells such as macrophages, endothelial cells, epithelial cells, and the abnormally high expression of TLRs is an important indicator of inflammation [48–51]. As a key upstream factor of TLR4/MyD88/NF-κB signaling pathway, extracellular leucine-rich repeat motif of TLR4 recognizes corresponding pathogen-associated molecular pattern or damage associated molecular pattern to form a dimer and binds to MyD88 through Toll/IL-1 receptor homologous region. Subsequently, the interaction of MyD88 and IL-1 receptor-associated kinase are formed, and activated TRAF6 resulting in the release into cytosol to trigger IKK complex, leading to activation and nuclear translocation of NF-κB, thereby promoting the release of downstream cytokines, such as TNF-α, IL-1β, IL-6 [52–55]. The inflammatory cytokines further activate NF-κB through positive feedback and produce a cascade reaction, which continuously exacerbates inflammatory injury. The activation of NF-κB plays a key role in the initiation and process of mucosal inflammation, affecting injury and repair of oral mucosa [45, 56, 57]. The results showed that QWS downregulated the expressions of TLR4, TRAF6, MyD88, p-IκBα and NF-κB p65 protein in oral mucosal tissues of OU subjected to stomach heat syndrome in db/db mice and downregulated the ratio of p-IκBα/IκBα, suggesting inhibited efficacy on TLR4/MyD88/NF-κB pathway, which contributes to compromise of inflammatory injury and eventually improvement of OU.

## Conclusion

In summary, we firstly demonstrated that QWS treatment effectively facilitated healing of OU, ameliorated pathological morphology of gastric and oral mucosa, and decreased the levels of pro-inflammatory cytokines in db/db mice subjected to stomach heat syndrome, whose mechanisms were associated with the suppression of TLR4/MyD88/NF-κB signal pathway. Our work provides experimental data for clinical application of QWS to treat diabetic OU subjected to stomach heat syndrome, and also contributes to scientific validation for indications intervention by classic Chinese Medicine prescriptions.

## Supplementary Information

**Additional file 1: Table S1.** The detailed information of active ingredients of QWS.

**Additional file 2: Fig. S1.** A. Common targets between OU and QWS. B-D. GO functional term enrichment analysis of (B) Biological process, (C) Cellular component and (D) Molecular function.

## Data Availability

The datasets used and analyzed during current study are available from the corresponding author on reasonable request.

## Abbreviations

QWS: Qingwei San
OU: Oral ulcer
T2DM: Type 2 diabetes mellitus
SH: Stomach heat syndrome
TXB_2_: Thromboxane B_2_
6-keto-PGF1α: 6-Keto-prostaglandin F1α
PGI_2_: Prostacyclin
IL: Interleukin
TNF-α: Tumor necrosis factor-α
β-EP: β-Endorphin
5-HT: 5-Hydroxytryptamine
TLR4: Toll-like receptor 4
TRAF6: TNF receptor associated factor 6
MyD88: Myeloid differentiation factor 88
IκΒα: Inhibitor of NF-κB alpha
NF-Κb: Nuclear factor kappa-B
PPI: Protein–protein interaction
GO: Gene ontology
KEGG: Kyoto Encyclopedia of Gene and Genome
BP: Biological process
MF: Molecular function
CC: Cellular component
