# Supplementary figures and images for: Qingwei San treats oral ulcer subjected to stomach heat syndrome in db/db mice by targeting TLR4/MyD88/NF-κB pathway
Source: Chin Med. 2022 Jan 4;17:1. doi: 10.1186/s13020-021-00565-5 (PMC8725453; doi:10.1186/s13020-021-00565-5)

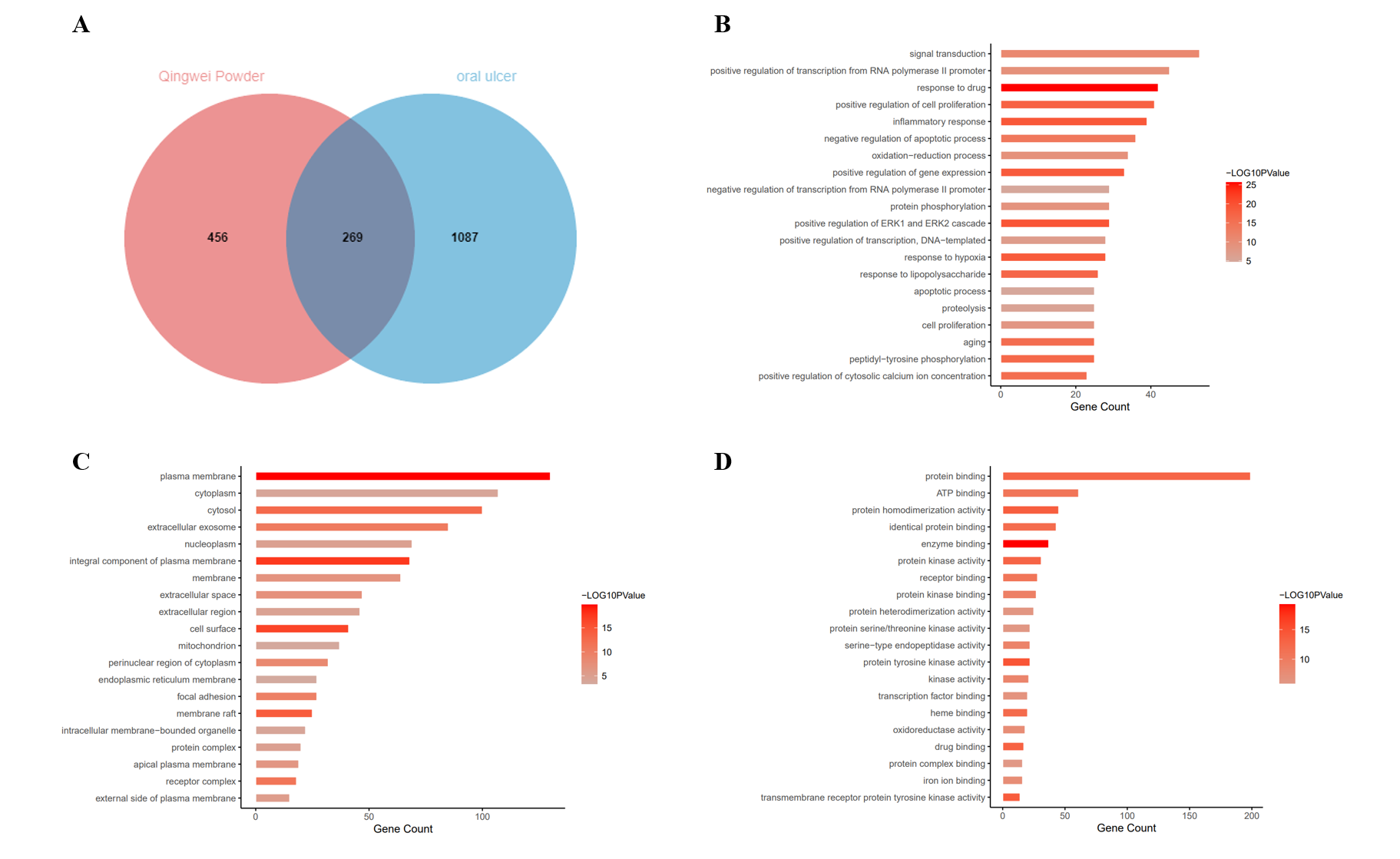

Supplement: Supplementary file 2 — Additional file 2: Fig. S1. A. Common targets between OU and QWS. B-D. GO functional term enrichment analysis of (B) Biological process, (C) Cellular component and (D) Molecular function. [file 13020_2021_565_MOESM2_ESM.tif]
